# The Dancing Mania

**Published:** 1914-05

**Authors:** 


THE DANCING MANIA.—Dancing has become so universal, so world-wide, that it is being classed as a craze or mania. People who dance the old square dances are now looked upon somewhat as antiques. The more modern waltz is regarded as a back number, and to be up-to-date one must engage in the bunny hug, the turkey trot, the tango or other of the "right-now" steps. The new dances differ from the dignified waltz in that they are wild, stimulating and emotional, that they have hurry and quick action characteristic of the present day. There is a difference of opinion as to whether the new dances tend more than the old to excite somatic sexual feelings and as to whether appetence is abnormally increased thereby; but there is no denial of the assertion that this psychic epidemic, since becoming a craze, is tending towards the impairment of health. Moderate dancing is not distinctly detrimental to health, but the new dances hardly allow of moderation. They seemingly make people "just crazy" for more and more of the same thing, and so we hear of "Tango-Foot" as a disease resulting from overdancing. What effect the epidemic will have upon the generation next to be born remains to be seen, but there is no reason to believe that it will be physically improved by the modern dance with its stimulating excitement?

